# Beneficial effects of physical exercise on the osteo-renal Klotho-FGF-23 axis in Chronic Kidney Disease: A systematic review with meta-analysis

**DOI:** 10.7150/ijms.90195

**Published:** 2024-01-01

**Authors:** Rafael Fernandez Castillo, Raquel García Pérez, Antonio Liñán González

**Affiliations:** 1 Instituto de Investigación Biosanitaria ibs.Granada; Faculty of Health Sciences, University of Granada, 18071 Granada, Spain.; 2 University of Granada. Faculty of Health Sciences, Parque Tecnológico de Ciencias de la Salud. Avd de la Ilustración 60 CP18016 Granada/Spain.

**Keywords:** Klotho protein, chronic kidney disease, Vitamin D, FGF23, training, exercise

## Abstract

The aim of this study was to investigate the efficacy of physical exercise in chronic kidney disease, describing its impact on the Klotho-FGF23 axis. PubMed, Web of Science and Scopus databases, updated to January 2023, were searched. The present study employed mean difference and a 95% confidence interval (CI) to examine the efficacy of the intervention. Heterogeneity was assessed through inconsistency statistics (I2). Out of the 299 studies identified, a total of 4 randomized controlled trials (RCTs), comprising 272 participants, met the eligibility criteria. Compared with the control group, physical exercise significantly decreased the concentrations of FGF23 (MD: -102.07 Pg/mL, 95% CI: -176.23.47, -27.91 I2= 97%, p = 0.001), and a significantly increased the concentrations of Klotho protein: (MD: 158.82 Pg/mL, 95% CI: 123.33, -194.31, I2 = 0%, p = 0.001). The results of our study indicated that the exercise has a direct relationship with Klotho-FGF23 axis. We can conclude that physical exercise in patients with CKD produces beneficial effects on the pathophysiological components related to this disease, including cardiorespiratory fitness and vascular functions. As observed, both endurance and aerobic physical exercise increase Klotho production and decrease FGF23 levels. Evidence indicates that exercise attenuates the progression of CKD, improves uremic parameters and down-regulates inflammation-related markers.

## Introduction

Chronic kidney disease (CKD) is a public health concern due to a very high prevalence (10% of the general population); it is expected to affect 9.1 million people by 2030 1 due to the growing incidence of type 2 diabetes mellitus, hypertension, obesity and aging 2. The main cause of mortality in CKD is cardiovascular (CV), with an increase in cardiovascular risk (CVR) 20 times higher than in the general population 3. It is estimated that approximately 11% of stage 3 CKD cases will progress to end-stage renal disease (ESRD) and eventually require dialysis or kidney transplantation 4. Eighty percent of patients with CKD present cardiovascular events, including arterial hypertension (36%), ischemic heart disease (22-39%), heart failure (20-40%), atrial fibrillation (30%), valvular heart disease (24%) and left ventricular hypertrophy (LVH), which occurs in 50-75% of patients with stage 3 or 4 CKD 5. It is therefore necessary to establish a clinical strategy in order to prevent the progression of CKD, including effective interventions to modify lifestyle risk factors. Lifestyle factors that can be modified to prevent the progression of CKD include obesity, smoking, sleep deprivation and physical inactivity 6-9.

Current therapeutic options to delay the progression of CKD are limited to a small handful of therapeutic options, such as renin-angiotensin-aldosterone system inhibitors, sodium bicarbonate supplementation, and sodium-glucose transporter 2 (SGLT-2) inhibitors 10,11. In the search for additional strategies, physical exercise has been considered as a possible treatment to delay disease progression 12.

**Figure 1:**
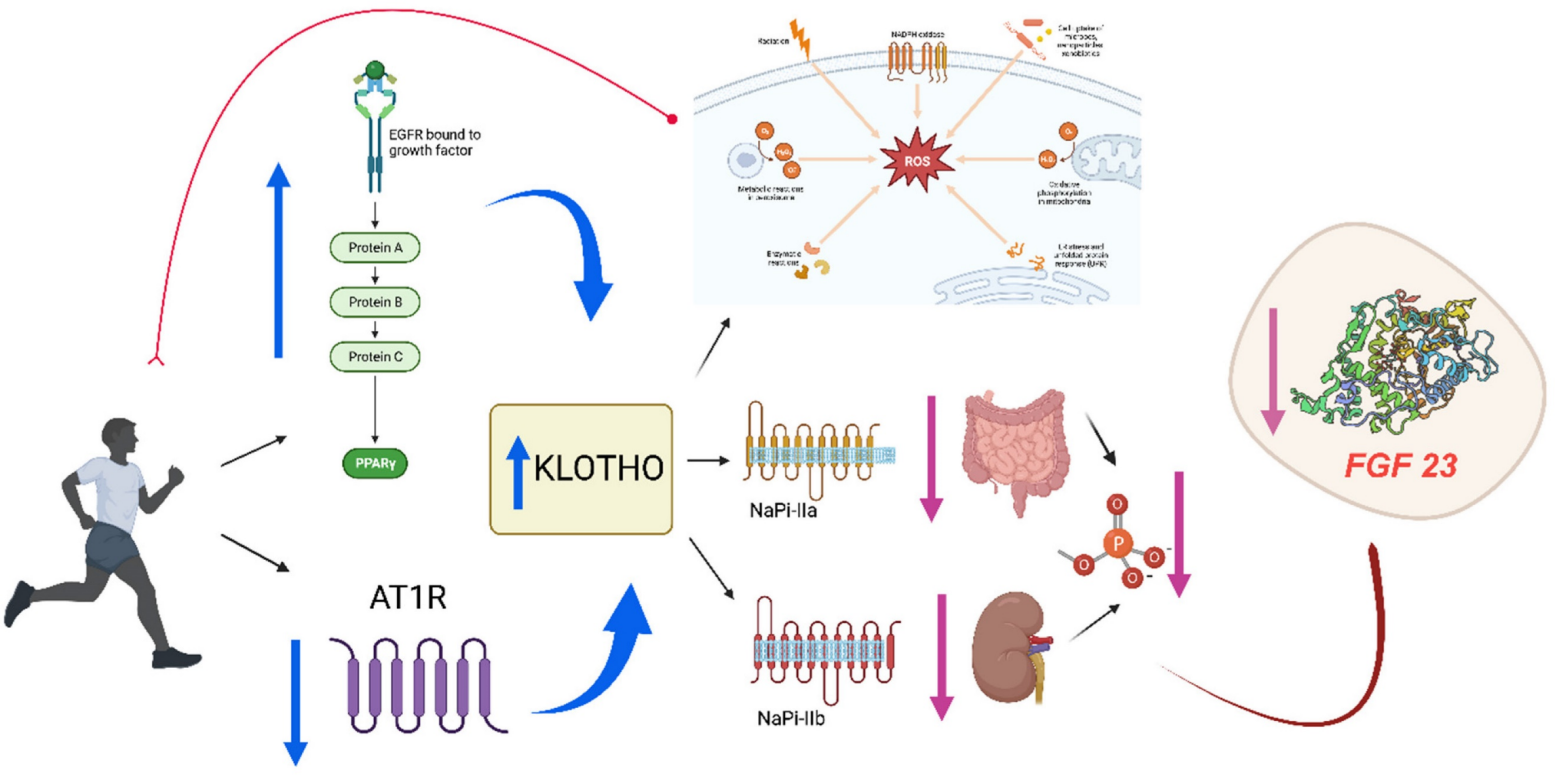
Exercise training might increase circulating Klotho due to increases in peroxisome proliferator-activated receptors (PPAR) and decreases in angiotensin II type I receptor (AT1R) signaling (10). On the other hand, physical exercise accelerates the production of reactive oxygen species (ROS) and Klotho is an important regulator of ROS through the activation of potent antioxidants such as catalase or superoxide dismutase, therefore the levels of the protein would increase fundamentally for the control of oxidative stress, but also high levels of this protein contribute directly to the process of cell regeneration and proliferation through an efficient call to the satellite cell, which also translates into a control of inflammation. Klotho protein maintains ion homeostasis by regulating ion channels and/or phosphate transporters. The high Klotho protein levels, as a result of exercise, inhibit renal and intestinal phosphate transport (NaPi-IIII. phosphate transportation (NaPi-IIa and NaPi-IIb) avoiding phosphate reabsorption and decreasing FGF23 levels.

Despite evidence of exercise-induced acute kidney injury in endurance athletes and increased proteinuria after exercise, concerns that exercise may have a detrimental effect on renal function have been allayed by a large number of studies showing that exercise is safe 13-15. In fact, structured exercise has been shown to have multiple beneficial effects in the CKD population, including improved aerobic capacity 16,17, physical function, muscle strength and health-related quality of life 18,19. Increased habitual physical activity is associated with improved survival in CKD patients by inducing an increase in Klotho protein 20-22 (Figure 1). This protein is related to human aging and to morbidity and mortality in patients with CKD; thus, an increase in the expression of this protein with exercise may be a positive prognostic factor for better outcomes in patients with CKD 23. On the other hand, patients with end-stage CKD may present fibroblast growth factor 23 (FGF23) values of up to 100 times their normal value 24. Elevated levels of FGF23 are associated with increased mortality due to cardiovascular risk factors and vascular calcifications. Klotho is a co-receptor of FGFR-1 and, therefore, facilitates the phosphaturic action of FGF23 25,26, inhibiting vascular calcification. For this reason, we conducted a systematic review of the evidence from randomized controlled trials (RCTs) investigating the efficacy of physical exercise in chronic kidney disease, describing its impact on the Klotho-FGF23 axis.

## Materials and Methods

This review and meta-analysis were carried out based on the guidelines proposed by PRISMA 27 (Preferred Reporting Items for Systematic Reviews and Meta-Analyses). We describe the methodology used to systematically present our findings on the efficacy of physical exercise in chronic kidney disease and its impact on the Klotho-FGF23 axis. The protocol for this study was registered in the PROSPERO database with the number “CRD42023441175”.

### Search Strategy

We systematically searched online medical databases, including Web of Science, PubMed/Medline, and SCOPUS for entries up to January 2023. The following search terms were used: (“exercise” OR “training” OR “aerobic” OR “randomized controlled trial”) AND (“CKD” OR “renal disease” OR “renal pathology”) AND (“Klotho” OR “α-Klotho” OR “FGF23”). Search equation descriptors were obtained from the thesaurus Medical Subproject headings (MeSH).

### Study Selection

Titles and abstracts of all articles obtained from the initial search were individually reviewed by the author. Studies published in Spanish and English and in the last 10 years were selected, in order to review the most current evidence; in this regard randomized trials, with a minimum duration of two weeks and analytical studies that evaluated the effects of exercise on chronic kidney disease and its impact on the Klotho-FGF23 axis were included. Studies that were not related to the subject or that did not provide relevant statistical information, research conducted on animals, systematic reviews or meta-analyses or uncontrolled experimental studies and research without a control group were excluded.

### Data Extraction

From each selected article, information was extracted including the name of the first author, the date of publication, the average age of the participants, and the gender of both the experimental and control groups, in addition to the study design, the participants, the duration, Intervention: exercise training; Comparator, no exercise group; Outcome: Klotho response. We also extracted the associated benefits related to the Klotho response in each study, and the means and standard deviations (SD) of both groups. Data related Klotho, and FGF23 were selected.

### Quality Assessment

Risk of bias assessment was conducted using the Cochrane method 28. The elements evaluated were the following: random sequence generation, allocation concealment, blinding of participants and personnel, blinding of outcome assessment, incomplete outcome data, selective reporting, and other bias.

Studies were classified as high risk of bias, low risk of bias, or unclear bias, for each item assessed, based on the recommendations of the Cochrane Handbook.

RevMan Web software (London, UK) was used to develop meta-analyses Meta-analyses were performed assessing the difference in post-intervention means, to estimate the heterogeneity of the included studies, the inconsistency statistic (I) was used, understanding heterogeneity as low if I^2^ < 50%, moderate if I^2^ 50-75% and high if I^2^ > 75%, with meta-analysis being recommended when the I^2^ is low or moderate. Publication bias was assessed using funnel plots.

## Results

**Figure 2:**
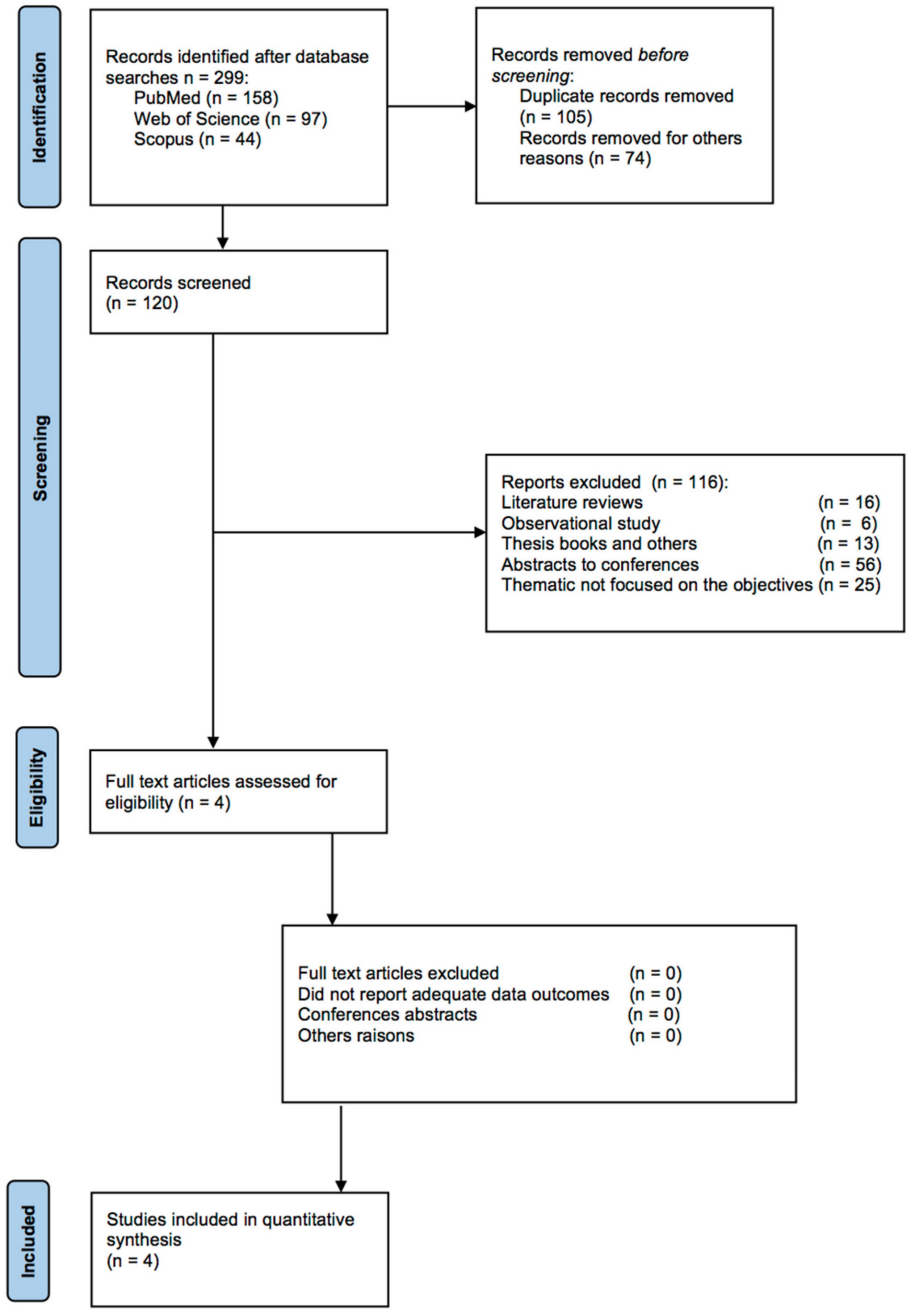
PRISMA flowchart of study selection.

The flow of studies in our meta-analysis is depicted in Figure 2, from 299 possibly relevant references. References were reviewed to determine eligibility. Finally, only four investigations met the eligibility criteria. These studies included 141 participants did some kind of physical exercise and 131 in control groups.

The characteristics of these four randomized controlled trials are presented in table 1.

From the analyzed studies, two presented the effects of resistance exercise in patients with stage 2 chronic kidney disease on the Klotho-FGF23 axis 29,30, maintenance of glomerular filtration rate (GFR) and bone mineral density (BMD). Two studies showed the effect of resistance exercise in patients with stage 5 chronic kidney disease on the Klotho-FGF axis, progression and attenuation of chronic kidney disease, iPTH and bone markers 31,32. None of the analyzed variables showed the existence of publication bias.

### Effects on KLotho

**Figure 3:**
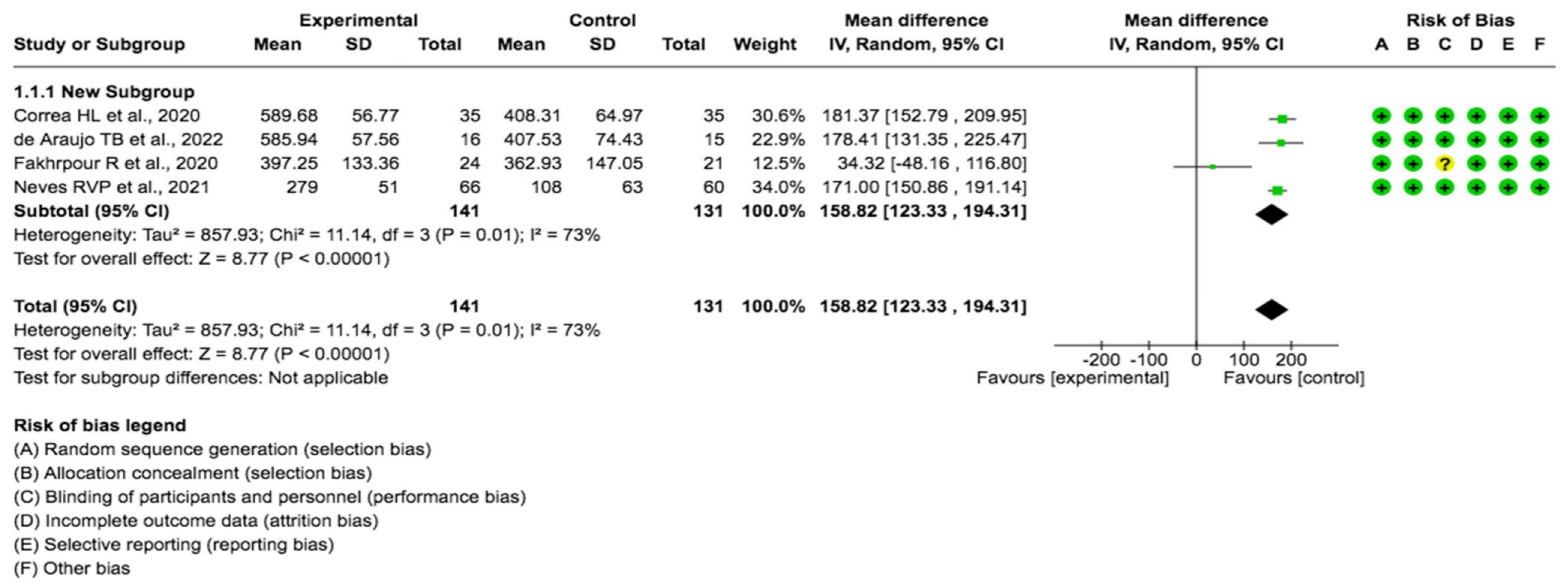
Forest plot for Klotho protein (29,30,31 32).

A pooled data analysis from 272 subjects and four studies showed that the exercise had a positive impact on Klotho levels, although positive effects were observed between the intervention and control groups. The mean difference and corresponding 95% confidence interval were 158.82 (122.33, 194.31) in favor of the control group, and the differences were considered significant (p= 0.001) (Fig. 3).

### Effects on FGF23

**Figure 4:**
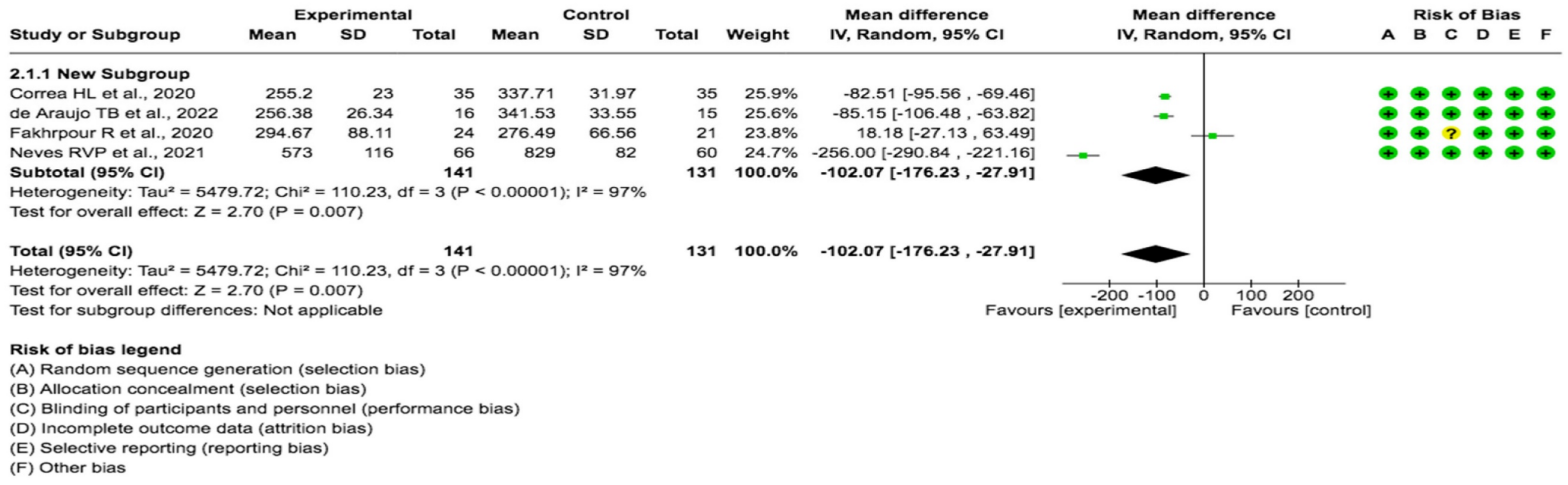
Forest plot for FGF23 (29,30,31 32).

A pooled data analysis from 272 subjects and four studies showed that the exercise had a positive impact on FGF23 levels, although positive effects were observed between the intervention and control groups. The mean difference and corresponding 95% confidence interval were -102.07 (-176.23, -27.91) in favor of the experimental group, and the differences were considered significant (p= 0.001) (Fig. 4).

## Discussion

The aim of this work was to investigate the efficacy of physical exercise in chronic kidney disease by describing its impact on the Klotho-FGF23 axis. The results of our study show that physical exercise has a direct relationship with an increase in Klotho levels as well as a reduction in FGF23 levels.

### Effects of physical exercise in CKD

Cardiovascular disease (CVD) is the leading cause of death in patients with end-stage CKD undergoing dialysis. As CKD progresses, the risk of cardiovascular morbidity and mortality also increases in these patients. Mortality is estimated at 56.4% in patients requiring chronic HD, of whom more than half die from CVD 33,34. These cardiovascular diseases are caused by vascular calcification and increased inflammatory and prothrombotic markers, leading to alterations in the intimal layer (endothelial dysfunction) and medial layer (arterial stiffness) of the blood vessel wall 34,35. Physical exercise has well-known beneficial effects on the heart, skeletal muscle and vascular wall. In addition to traditional risk factor modification, exercise improves vascular health through increased nitric oxide bioavailability as well as generalized antioxidant and anti-inflammatory effects. As such, regular physical activity is strongly recommended 36. In hemodialysis patients, a twice-weekly aerobic exercise program for 3 months was suggested to significantly improve the aortic augmentation index (AIx) and aortic pulse wave velocity (both markers of arterial stiffness), which returned to baseline levels within 1 month after cessation of training 37.

Exercise has also been shown to be effective in reducing systolic and diastolic blood pressure (BP), which represents a high cardiovascular risk in subjects with CKD. A meta-analysis revealed that the combination of aerobic and resistance training, as well as the application of high-intensity aerobic training, have a superior effect on BP reduction 38.

Another important factor is bone abnormalities, which are a major cause of morbidity and decreased quality of life in CKD patients 39.

Bone disease in HD patients is mainly due to the effect of secondary hyperparathyroidism (SHPT). Dialysis patients develop bone resistance to the action of PTH and therefore need higher levels to achieve normal bone remodeling. If patients present chronically elevated PTH levels, they develop osteitis fibrosa (high-bone-remodeling disease); on the contrary, if PTH levels remain normal, this is associated with adynamic bone disease (low-bone-remodeling disease) 40.

Repetitive stress applied to weight-bearing sites over long periods of time strengthens bones; thus, both high- and low-intensity resistance training can change the biochemical rates of bone turnover. In addition, strength training can regionally increase bone mineral density (BMD) by increasing bone formation. Endocrine regulators of bone, such as PTH, vitamin D and calcium, may also be affected during periods of strenuous exercise and may enhance bone metabolism. A study by Marinho et al. suggests that a 24-week intradialytic resistance exercise program is effective in improving BMD, especially in the femoral neck, leading to a slight reduction in PTH levels (clinically significant) 39. According to our results, strength and aerobic exercise attenuated the decrease in glomerular filtration rate (GFR), with the majority of patients improving to CKD stage 3 (88.5%) and showing improved uremic parameters, functional capacity, bone mineral density and immunometabolic profile 30-32 as well as decreased PTH 31 and inflammation 29. The effects of physical exercise in general are undisputed in CKD; however, despite the positive impact on risk factors for disease progression, the effect of exercise on renal function per se is unclear. The divergent results of recent meta-analyses (including ours) reflect the heterogeneity of the method of analysis and of the included studies, as well as the heterogeneity of the individual study designs, especially in terms of duration, intensity of the interventions and uncertainty regarding participants' compliance with the exercise program, preventing conclusive results [15.20,41,42].

### Effects on Klotho protein

As previously mentioned, Klotho protein levels decrease in the early stages of CKD^43^. Its deficit could lead to vascular calcification by promoting the entry of P into VSMCs, arteriosclerosis, osteoporosis, ectopic calcification, premature aging, apoptosis and the progression of CKD 43,44. Its suppression also implies decreased phosphaturia, with increased P and serum calcitriol levels. In experimental models, a decrease in Klotho has been associated with acute renal failure 45, suggesting its role as a possible biomarker. Likewise, its replenishment could lead to the recovery of renal damage 46.

Physical activity also induces an increase in the αKlotho (αKl) protein 47. This protein is encoded by the Klotho gene, which is related to human aging and to morbidity and mortality in patients with CKD. Thus, the increased expression of this protein with exercise may be a positive prognostic factor for limiting disease progression 48. In general, the increase in Klotho improves renal fibrosis and recovery from acute kidney injury and reduces hypertension 49. In addition, increased Klotho expression has also been shown to improve endothelial function 50, reduce vascular calcification 51, and improve uremic cardiomyopathy in CKD models 52. Thus, an increase in Klotho expression benefits organ systems that are integral to physical function, including the heart, the vascular system and, of course, skeletal muscle. In our review, we observed that physical training seems to stimulate S-Klotho independently of the protocol (resistance or aerobic training) 53. However, depending on the type of intervention, we observed some differentiation in studies with resistance and aerobic training. Of the studies with resistance training 29-32, all applied full-body resistance training, using exercises that require several muscle groupings in the same movement. et al. 30 applied a total of twenty exercises, Neves et al. 32 used a total of twelve exercises (for both dynamic and isometric training), Corrêa et al. 29 used eight exercises and Fakhrpour et al. 31 applied a total of five exercises. Although the final exercise load was similar in the included studies, it is known that different training configurations, such as number of sets and repetitions, produce different metabolic responses in humans 54. Considering that Klotho expression is mainly influenced by metabolism 55,56, it is possible that different strength exercise prescriptions may lead to different S-Klotho responses. For the studies that applied aerobic training, one study used a treadmill for intervention 31. We observed differences in the Klotho data between this and the other interventions 19,20,32. This could be due to the impact of aerobic exercise stimulation on mitochondrial biogenesis 57. As noted above, oxidative metabolism can influence Klotho production. Therefore, aerobic training induces S-Klotho due to the overall mitochondrial stimulation. Thus, a combination of aerobic and resistance training is the optimal protocol to increase S-Klotho in humans.

### Effects on FGF23

Patients with end-stage CKD may have FGF23 values up to 100 times the normal value 58. Furthermore, high levels of FGF23 predict CKD progression, as has been confirmed in numerous studies 59-61. Elevated FGF23 levels are associated with increased mortality as adjusted for classical cardiovascular risk factors and other traditional markers of CKD 62-64. This bone-derived protein increases urinary phosphate excretion and reduces calcitriol (i.e., the active form of vitamin D) levels. However, as the GFR in the kidney decreases, phosphate accumulation in the blood increases, which in turn leads to a reduction in calcitriol production and also stimulates increased secretion of parathyroid hormone, leading to hyperparathyroidism 65.

As we observed in our results, both endurance and aerobic training attenuate the decrease in GFR and improve uremic parameters, disease progression and inflammation, by down-regulating proinflammatory cytokines and increasing anti-inflammatory cytokines. The FGF23-Klotho axis is improved in patients who participated in both exercise models, with an increase in Klotho concentration and a decrease in FGF23 29-32; this is due to the increase in central vascular blood flow and vascular permeability caused by exercise, which provides a greater exchange area between intracellular (where we find phosphorus) and intravascular compartments. Therefore, the renoprotective effect of both training models through the negative regulation of FGF23 and the increase in Klotho concentrations seems clear.

### Limitations

This study has some limitations, the number of studies included in the meta-analysis was low. Thus, the results should be treated with caution and future studies should perform a similar and protocolized exercise intervention. Thus, more research should be done focusing on this topic in the future.

## Conclusion and future directions

We can conclude that physical exercise in patients with CKD produces beneficial effects on the pathophysiological components related to this disease, including cardiorespiratory fitness and vascular functions. As observed, both endurance and aerobic physical exercise increase Klotho production and decrease FGF23 levels. Evidence indicates that exercise attenuates the progression of CKD, improves uremic parameters and down-regulates inflammation-related markers.

The favorable effect of exercise on serum concentrations of Klotho and FGF-23 may be related to better control of oxidative stress, chronic inflammation and metabolic acidosis that develops as a result of energy wasting.

However, despite the benefits on risk factors for disease progression, the effect of exercise on renal function is unclear. The mixed results of meta-analyses (including this one) reflect the heterogeneity of the methods of analysis and of the included studies. Furthermore, the heterogeneity of the study designs, especially with regard to the duration and intensity of the exercise interventions, precludes definitive conclusions. Moreover, uncertainty regarding participants' adherence to the exercise programs also hinders the assessment of efficacy.

Despite notable advances in Klotho research, several gaps remain in our knowledge of its role and mechanisms in skeletal muscle. For example, the distinct functional roles of S-Klotho isoforms in skeletal muscle and their molecular mechanisms are unknown. In addition, the precise concentrations of S-Klotho that are necessary to maintain or improve physical function are unknown and are likely dependent on genetic variability between populations. Further research is needed to determine the role of Klotho levels in muscle function in various stages of CKD and other disease populations. As verified in the present study, exercise appears to modify the Klotho-FGF23 axis in patients with CKD. Elucidating the mechanisms of Klotho signaling in skeletal muscle may provide additional opportunities for the development of treatments that can address the increasing burden of sarcopenia and its progression to physical disability.

In this regard, it is important to map the benefits of exercise in CKD patients and compare the Klotho and FGF23 release profile with healthy subjects. This may allow the development of databases elucidating the Klotho-FGF23 axis following physical exercise in healthy versus CKD patients. This would facilitate the design of further studies with the aim of identifying exercise mimetics for those who have physical impairments that preclude the practice of physical exercise.

## Figures and Tables

**Table 1:** Characteristics and summary of the studies included in the review.

Reference	Study Type	Subjects (*n*) and Characteristics	Exercise	Age (years)	Main Outcomes	Associated benefits	Country
Corrêa et al., 2021 29	RCT double-blind cross-over study 6 month. Frequency: 3 days/week	*n* = 105, 72men and 33 women.Stage 2 CKD	Resistance Training (RT)	58 ± 5	↑ Klotho 30.7% ↓ FGF23 25.3%	Attenuated the progression of the disease by maintaining GFR, improving uremic parameters, cytokine profile regulation, and klotho-FGF23 axis	Brazil
De Araujo et al., 2015 30	RCT double-blind cross-over study 22 weeks. Frequency: 3-4 days/week	*n = 31.*Stage 2 CKD	Home-based RT	58.07 ± 5.22	↑ Klotho 17.6% ↓ FGF23 1%	Attenuatd the progression of CKD and improve functional capacity, bone mineral density, and the immunometabolic profile. Positive modulation of several exerkines.	Brazil
Fakhrpour R 2020 et al., 31	RCT double-blind cross-over study 16 weeks. Frequency: 3 days/week	*n = 45Stage 5 CKD dialysis*	Combined aerobic and RTexercise program during dialysis	61 ± 9.02	↑ Klotho 10% ↓ FGF23 7.3%	Improves quality of life and physical functions. Reduction of iPTH and phosphorous levels were observed in treated patients.	Iran
Neves RVP et al., 2021 32	RCT double-blind cross-over study 24 weeks. Frequency: 3 days/week	*n = 193Stage 5 CKD dialysis*	Conventional RT	56.3 ± 5	↑ Klotho 88.51% ↓ FGF23 30 %	Improves bone mineral biomarkers and bone mineral density	Brazil
